# CDSS score is favorable to ISTH score on outcomes for disseminated intravascular coagulation in patients with liver transplantation: a retrospective cohort study

**DOI:** 10.3389/fmed.2025.1514139

**Published:** 2025-04-30

**Authors:** Li Zhong, Yan Liu, Conglin Wang, Lei Su, Zhifeng Liu, Ming Wu

**Affiliations:** ^1^Department of Medical Critical Care Medicine, General Hospital of Southern Theatre Command of Peoples Liberation Army, Guangzhou, China; ^2^Department of Traditional Chinese Medicine, The First Affiliated Hospital, Guizhou University of Chinese Medicine, Guiyang, China; ^3^Department of Infection and Critical Care Medicine, Shenzhen Second People’s Hospital & First Affiliated Hospital of Shenzhen University, Shenzhen, China; ^4^Department of Nosocomial Infection Prevention and Control, Shenzhen Second People’s Hospital, Shenzhen, China; ^5^Guangdong Branch Center, National Clinical Research Center for Geriatric Diseases (Chinese PLA General Hospital), Guangzhou, China; ^6^Key Laboratory of Hot Zone Trauma Care and Tissue Repair of Peoples Liberation Army, General Hospital of Southern Theatre Command of Peoples Liberation Army, Guangzhou, China; ^7^Department of Critical Care Medicine, Shenzhen Guangming District People's Hospital, Shenzhen, China

**Keywords:** liver transplantation, DIC, CDSS score, ISTH score, outcome

## Abstract

**Background:**

Limited data are available regarding disseminated intravascular coagulation (DIC) scores after liver transplantation (LT). As Chinese DIC Scoring System (CDSS) is widely accepted for assessing coagulation in China, this study was aimed to investigate the prognostic value of CDSS scores in patients with undergoing LT.

**Method:**

A retrospective cohort study was conducted on patients who underwent LT from November 2009 to October 2021. We validated CDSS criteria by comparing with International Society on Thrombosis and Hemostasis (ISTH) score. Additionally, its prognostic value was evaluated with receiver operating characteristic (ROC) curves and odds ratio based on mortality rates at 28, 60, and 90 days, as well as the correlations between the CDSS score and acute physiological and chronic health assessment II (APACHE II), sequential organ failure assessment (SOFA) scores at 90-day mortality.

**Results:**

A total of 569 LT patients were enrolled, of which 80 patients developed DIC with CDSS score and 305 patients with ISTH score. Patients with DIC using the CDSS exhibited higher APACHE II and SOFA scores than those with ISTH score. The incidences of acute kidney injury, infection, lymphocytopenia and mortality were higher in DIC patients with CDSS than in those with ISTH. When assessing the prognostic value for 28-day mortality, the CDSS demonstrated higher sensitivity (64.61% vs. 50.77%), but lower specificity (73.62% vs. 88.89%) compared to the ISTH, the areas under ROC (AUC) for the CDSS and ISTH scores were 0.739, 0.741 (*p* < 0.05) and the odds ratios (OR) for the CDSS and the ISTH were 6.228, 3.597, respectively (*p* < 0.05). The ORs for predicting mortality with 60-day (7.719 vs. 3.95) and 90-day (7.582 vs. 3.95) criteria with CDSS were higher than those with ISTH (*p* < 0.05). The Spearman’s rank correlation coefficients between the CDSS and APACHE II scores, and the SOFA scores were 0.217 and 0.422, respectively, compared to 0.19 and 0.371 for the ISTH score (*p* < 0.001).

**Conclusion:**

Disseminated intravascular coagulation presents a life-threatening complication in perioperative period of LT. The CDSS score has better prognostic value than the ISTH score for DIC patients after LT. A prospective randomized controlled study should be designed to further evaluate the findings.

## Introduction

Liver transplantation (LT) is a life-saving therapy for patients with acute and chronic liver disease at the end stage (1). The liver, as an important organ responsible for regulating coagulation, synthesizes and metabolizes most coagulation factors, including both pro-coagulation factors and anti-coagulation factors. The normal function of the coagulation system is crucial for maintaining the balance of circulation in the body (1–4). Coagulation dysfunction is common during the perioperative period of LT, and it involves various pathophysiological mechanisms, including coagulation promotion, anticoagulation, and fibrinolysis, at each stage. Abnormal coagulation function plays a critical role in the postoperative recovery and prognosis of patients undergoing LT (5–8). Evaluating coagulation in the Asia-Pacific region, especially in China, presents significant challenges for liver surgeons due to the race-specific characteristics of the clotting system (9–11).

The International Society of Thrombosis and Hemostasis (ISTH) score is widely acknowledged as a standard indicator for assessing coagulation dysfunction worldwide. In China, the Chinese disseminated intravascular coagulation (DIC) Scoring System (CDSS) is predominantly used (12, 13). However, there is limited data on the perioperative DIC scoring for patients undergoing LT. To fill this research gap, we collected preoperative and postoperative data of LT patients who were admitted to the department of intensive care medicine over the past 12 years and we evaluated the diagnostic and prognostic significance of the CDSS and ISTH scores in LT patients.

## Materials and methods

### Study design and participants

We conducted a retrospective cohort study using data from hospitalized patients who underwent liver transplantation (LT) at the General Hospital of Southern Theatre Command of the People’s Liberation Army Hospital from November 2009 to October 2021. The study was approved by the Research Ethics Committee of our hospital under the approval number (NZLLKZ2022020). As the data collected in this study were retrospective and de-identified, the Ethics Committee waived the requirement for patients’ written informed consent. Inclusion criteria were established for patients who were 18 years of age or older and had undergone liver transplantation. The exclusion criteria included: (1) Patients who underwent liver, kidney, liver-lung combined transplantation, or combined transplantation involving other organs; (2) Patients who underwent non-total liver transplantation.

### Research procedures

The data collected by our research subjects were collected within 24 h after admission and within 24 h after the first postoperative admission to the ICU for statistical analysis. We collected demographic and clinical data from the patients, including gender, age, Acute Physiology and Chronic Health Evaluation II (APACHE II) score, Sequential Organ Failure Assessment (SOFA) score, Model for End-Stage Liver Disease (MELD) score, coagulation parameters include fibrinogen (FIB), prothrombin time (PT), international normalized ratio (INR), activated partial thromboplastin time (APTT), thrombin time (TT), platelet index, and dates of hospital and ICU admissions. Additionally, we documented complications such as acute kidney injury (AKI), infection, and lymphocytopenia after liver transplantation. Liver transplantation patients were divided into DIC and non-DIC groups based on their CDSS and ISTH scores, respectively.

The clinical characteristics of patients were categorized by two scoring systems. We analyzed the sensitivity and specificity of the CDSS and ISTH scores in predicting 28-day, 60-day, and 90-day mortality. Receiver operating characteristic (ROC) curves were used to evaluate the prognostic accuracy of the CDSS and APACHE II scores for mortality at 28-day, 60-day, and 90-day time intervals. Additionally, we performed separate examinations on the correlations between the CDSS and ISTH scores and the APACHE II and SOFA scores.

### Definitions

DIC: Disseminated Intravascular Coagulation (DIC) is a clinical syndrome caused by various pathological factors that damage the microvascular system. It leads to the activation of coagulation, systemic microvascular thrombosis, consumption of coagulation factors, and secondary hyperfibrinolysis, resulting in bleeding and failure of microcirculation (8).

DIC diagnosis based on ISTH integration (9): The scoring system includes Prothrombin time (PT), Platelet count (PLT), Fibrinogen level (Fib), and fibrin-related markers (D-Dimer and Fibrinogen Degradation Products). A score of≥5 points indicates the diagnosis of DIC (Supplementary Table S1).

Disseminated Intravascular Coagulation diagnosis based on CDSS integral (8): The scoring system includes three components: underlying disease (presence of a primary cause of DIC), clinical manifestations (severe or multiple unexplained bleeding tendencies, microcirculatory disturbance or shock, generalized skin and mucosal embolism, focal avascular necrosis, exfoliation and ulcer formation, unexplained organ failure of lung, kidney, brain, and other organs), and laboratory indicators (platelet count, D-Dimer, PT, Activated partial thromboplastin time, and fibrinogen). A score of ≥7 indicates the diagnosis of DIC (Supplementary Table S1).

AKI (14): AKI was defined as an increase in serum creatinine ≥ 0.3 mg/dL (≥26.5 μmol/L) and within 48 h (or) serum creatinine increased to ≥1.5 times basal value and/or urine volume <0.5 mL/(kg·h) within 1 week and continued for 6 h.

### Statistical analysis

Measurements were expressed as median and 25th to 75th percentiles (Q1–Q3) for continuous variables. Comparisons between two groups were made using Mann–Whitney’s *U*-test. Proportions were compared by the chi-square test, or Fisher’s exact test when necessary. As there is no gold standard for diagnosing DIC, we constructed Receiver Operating Characteristic (ROC) curves to assess the prognostic prediction of each criterion. These ROC curves compared the ability of the CDSS and ISTH scoring systems to predict 28-day, 60-day, and 90-day all-cause mortality. The predictive ability of the CDSS and ISTH DIC score for mortality was further verified by the logistic regression analysis, with outcome as the criterion variate, and age, gender and DIC score as the explanatory variates. Correlations of the CDSS and the ISTH score with the APACHE II score and the SOFA score were examined according to the Spearman’s rank correlation coefficient. R-4.0.2 (R Foundation for Statistical Computing, Vienna, Austria) was used for statistical calculations and analyses. For all reported results, *p* < 0.05 was considered to be statistically significant.

## Results

### Demographics and baseline characteristics in patients with LT

**Figure 1:**
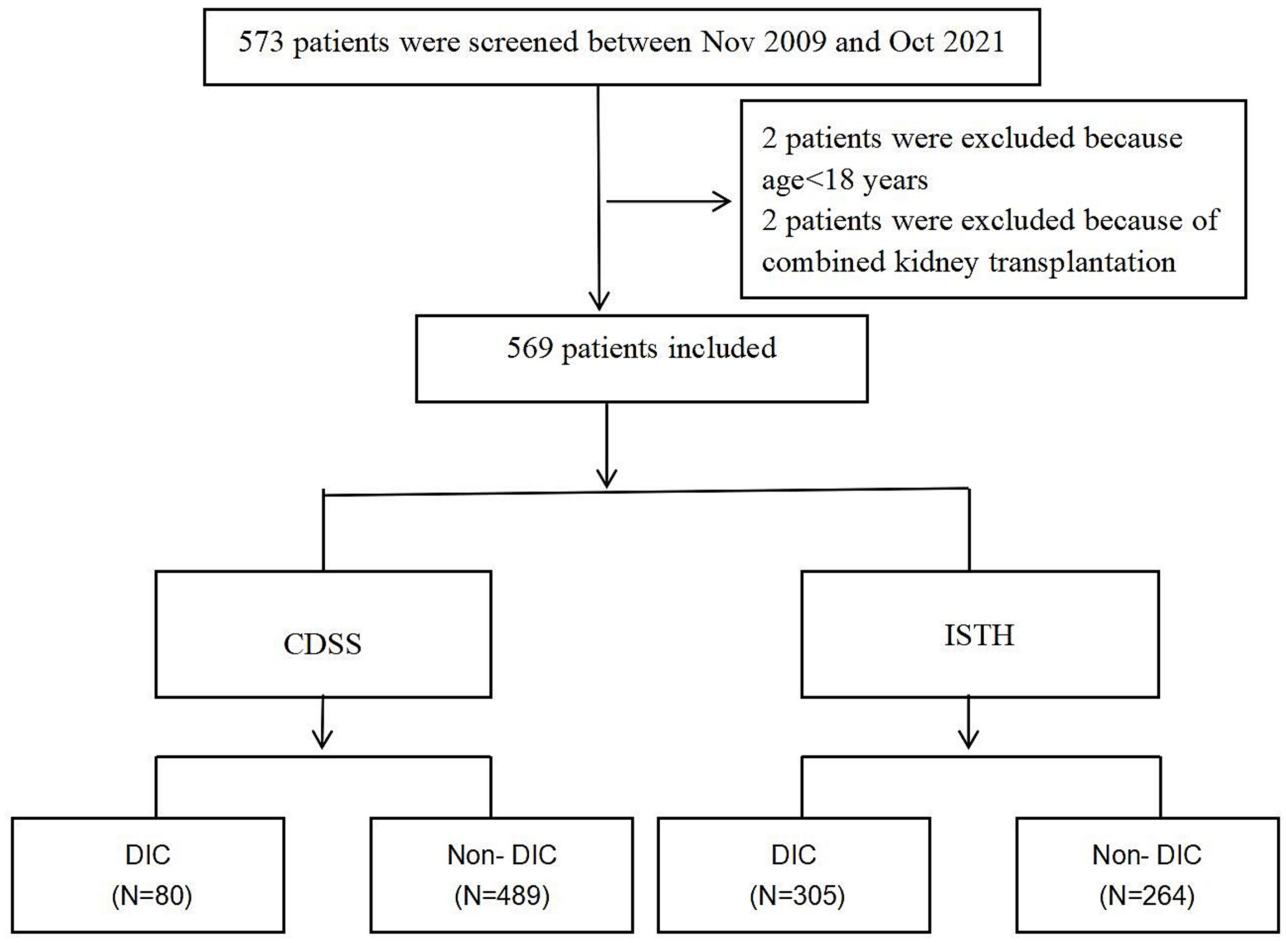
Flowchart of all excluded and included patients.

After applying the preliminary exclusion criteria, a total of 569 patients were included in the study. The top three causes of liver transplantation in our included patients were hepatocellular carcinoma (47.7%), decompensation of cirrhosis (38.4%), liver failure (12.6%), and other (1.3%). Among the patients included in the study, 80 cases (14.06%) were diagnosed, while 489 cases (85.9%) were not diagnosed with DIC with DIC with CDSS criterion. Among the patients diagnosed with DIC, 15 were females (18.8%), while 65 were males (81.2%). In the non-DIC group, there were 56 females (11.5%) and 433 males (88.5%) (Figure 1).

ISTH scores were used for the diagnosis of DIC. Among them, 305 cases (53.6%) were confirmed as DIC, while 264cases (46.4%) were not. In the DIC group, there were 43 cases (14.1%) of females and 262 cases (85.9%) of males. In the non-DIC group, there were 28 females (10.6%) and 236 males (89.4%) (Table 1).

**Table 1:** Baseline characteristics of the patients.

	Total (*n* = 569)	CDSS score		ISTH score	
		Non-DIC (*n* = 489)	DIC (*n* = 80)	Non-DIC (*n* = 264)	DIC (*n* = 305)
Gender (*n* %)					
Female	71 (12.5)	56 (11.5)	15 (18.8)	28 (10.6)	43 (14.1)
Male	498 (87.5)	433 (88.5)	65 (81.2)	236 (89.4)	262 (85.9)
Age (years) (mean ± SD)	50 ± 10	50 ± 10	49 ± 11	49 ± 10	50 ± 11
WBC (1 × 10^9/L) (median [Q1, Q3])	5.92 [4.04, 8.25]	6.05 [4.15, 7.82]	5.77 [3.89, 9.12]	5.89 [4.04, 7.97]	6.57 [4.05, 10.05]
Neutrophile (1 × 10^9/L) (median [Q1, Q3])	3.85 [2.36, 6.11]	3.83 [2.42, 5.45]	3.89 [2.28, 7.11]^a^	3.79 [2.33, 5.69]	4.76 [2.57, 7.88]
Lymphocyte (1 × 10^9/L) (median [Q1, Q3])	1.01 [0.67, 1.56]	1.16 [0.75, 1.72]	0.91 [0.58, 1.43]^a^	1.03 [0.70, 1.58]	0.86 [0.52, 1.43]^a^
Monocyte (1 × 10^9/L) (median [Q1, Q3])	0.46 [0.32, 0.69]	0.45 [0.32, 0.65]	0.47 [0.32, 0.76]	0.46 [0.32, 0.67]	0.51 [0.30, 0.88]
Eosinophilic granulocyte (1 × 10^9/L) (median [Q1, Q3])	0.09 [0.03, 0.17]	0.10 [0.04, 0.17]	0.07 [0.03, 0.16]^a,b^	0.09 [0.04, 0.18]	0.06 [0.02, 0.11]^b^
Basophilic granulocyte (1 × 10^9/L) (median [Q1, Q3])	0.02 [0.01, 0.03]	0.02 [0.01, 0.03]	0.02 [0.01, 0.03]	0.02 [0.01, 0.03]	0.01 [0.01, 0.03]
Hemoglobin (g/L) (median [Q1, Q3])	113 [90, 135]	117 [93, 137]	96 [76, 119]^a^	126 [100, 142]	105 [84, 125]
Ammonia (μg/L) (median [Q1, Q3])	55 [42, 75]	54 [41, 73]	65 [48, 100]^a^	51 [40, 66]	60 [44, 81]^a^
ALT (U/L) (median [Q1, Q3])	38 [25, 71]	39 [25, 68]	34 [23, 80]	39 [25, 67]	38 [24, 74]
AST (U/L) (median [Q1, Q3])	51 [34, 100]	49 [34, 92]	66 [36, 127]	43 [31, 86]	59 [37, 118]^a^
ALB (U/L) (median [Q1, Q3])	38 [33, 43]	38 [34, 43]	37 [33, 40]	40 [35, 44]	37 [32, 42]^a^
ALP (U/L) (median [Q1, Q3])	110 [77, 167]	112 [78, 171]	100 [73, 134]	112 [77, 174]	109 [78, 156]
Cr (μmol/L) (median [Q1, Q3])	73 [61, 88]	73 [62, 87]	74 [58, 96]	71 [62, 83]	76 [60, 94]^a^
Platelets (1 × 10^9/L) (median [Q1, Q3])	93 [54, 161]	103 [56, 168]	68 [38, 108]^a^	127 [76, 192]	71 [45, 118]^a^
FIB (g/L) (median [Q1, Q3])	2.7 [1.8, 3.7]	2.8 [1.9, 3.8]	1.8 [1.2, 2.7]^a,b^	3.2 [2.4, 4.2]	2.1 [1.5, 3.1]^a,b^
PT(s) (median [Q1, Q3])	15.4 [13.7, 19.3]	15.1 [13.6, 18.5]	19.1 [15.4, 25.0]^a,b^	14.2 [13.3, 16.0]	17.4 [14.6, 22.2]^a,b^
APTT(s) (median [Q1, Q3])	41.7 [36.8, 48.7]	40.8 [36.5, 47.2]	49.1 [41.9, 68.5]^a,b^	39.0 [35.6, 43.8]	44.6 [38.9, 53.9]^a,b^
TT(s) (median [Q1, Q3])	18.0 [16.9, 19.9]	17.9 [16.9, 19.5]	19.2 [17.0, 23.0]^a^	17.7 [16.8, 18.7]	18.6 [17.1, 21.0]^a^
Hematocrit (%) (median [Q1, Q3])	0.34 [0.27, 0.40]	0.35 [0.28, 0.41]	0.28 [0.23, 0.35]^a,b^	0.37 [0.31, 0.43]	0.31 [0.25, 0.37]^a,b^
INR (median [Q1, Q3])	1.22 [1.07, 1.64]	1.19 [1.05, 1.55]	1.69 [1.24, 2.35]^a,b^	1.10 [1.02, 1.29]	1.42 [1.15, 2.00]^a,b^
D-Dimer (mg/L) (median [Q1, Q3])	6.00 [3.24, 10.00]	4.94 [2.94, 8.58]	13.13 [9.51, 20.00]^a,b^	3.78 [2.63, 6.76]	7.76 [4.57, 12.18]^a,b^
PCT (ng/mL) (median [Q1, Q3])	0.42 [0.06, 2.36]	0.37 [0.06, 2.52]	0.68 [0.14, 1.75]^a^	0.27 [0.05, 2.00]	0.60 [0.08, 2.56]^a^
Cystatin C (mg/L) (median [Q1, Q3])	1.10 [0.90, 1.46]	1.08 [0.90, 1.39]	1.27 [0.94, 1.79]^a^	1.00 [0.84, 1.21]	1.23 [0.98, 1.65]^a^
TBIL (μmol/L) (median [Q1, Q3])	32.6 [14.8, 104.9]	29.0 [13.8, 79.0]	122.9 [33.9, 343.3]^a^	20.5 [12.0, 46.5]	53.3 [21.6, 233.5]^a^
APACHEII score (median [Q1, Q3])	9 [7, 12]	9 [7, 11]	12 [10, 15]^a,b^	9 [6, 11]	10 [8, 14]^a,b^
SOFA score (median [Q1, Q3])	7 [5, 9]	6 [5, 8]	9 [7, 12]^a,b^	6 [4, 7]	8 [6, 10]^a,b^
MELD score (median [Q1,Q3])	14 [9, 23]	13 [8, 21]	22 [13, 33]^a^	10 [8, 17]	17 [11, 30]^a^

WBC, white blood cell count; ALT, alanine aminotransferase; AST, aspartate aminotransferase; ALB, albumin; ALP, alkaline phosphatase; CR, creatine; FIB, fibrinogen; PT, prothrombin time; INR, international normalized ratio; APTT, activated partial thromboplastin time; TT, thrombin time; TBIL, total bilirubin; PCT, procalcitonin; DIC, disseminated intravascular coagulation; CDSS, Chinese DIC Scoring System; ISTH, International Society on Thrombosis and Hemostasis; APACHE, Acute Physiology and Chronic Health Evaluation; SOFA, sequential organ failure assessment. ^a^*p* < 0.05 between DIC and non-DIC; ^b^*p* < 0.05 between CDSS DIC and ISTH DIC.

### Comparison of groups between CDSS scores and ISTH scores

In the category based on the CDSS score, patients with DIC had higher APACHE II scores [12.0 (10.0–15.0) vs. 9.0 (7.0–11.0)], SOFA scores [9.0 (7.0–12.0) vs. 6.0 (5.0–8.0)] and MELD scores [22.0 (13.0–33.0) vs. 13 (8.0–21.0)] compared to patients without DIC. Additionally, patients in the DIC group exhibited more severe coagulation injury and a higher likelihood of co-infection, resulting in increased mortality rates at 28 days (32.5% vs. 7.2%), 60 days (43.8% vs. 9.0%), and 90 days (45% vs. 9.6%). Furthermore, hospital stay was prolonged and survival time was shortened in the DIC group (*p* < 0.05) (Table 1).

In the category based on ISTH guidelines, patients with DIC in the ISTH-based category had higher APACHE II scores [10.0 (8.0–14.0) compared to 9.0 (6.0–11.0)], SOFA scores [8.0 (6.0–10.0) compared to 6.0 (4.0–7.0)], and MELD scores 17.0 (11.0–30.0) compared to 10 (8.0–17.0) when compared to patients without DIC. Additionally, patients in the DIC group had a higher incidence of severe coagulation injury and co-infections, leading to increased mortality at 28 days (15.7% compared to 4.9%), 60 days (20.3% compared to 6.4%), and 90 days (21.6% compared to 6.4%). Furthermore, they experienced prolonged hospital stays and shortened survival times (*p* < 0.05) (Table 1).

**Figure 2:**
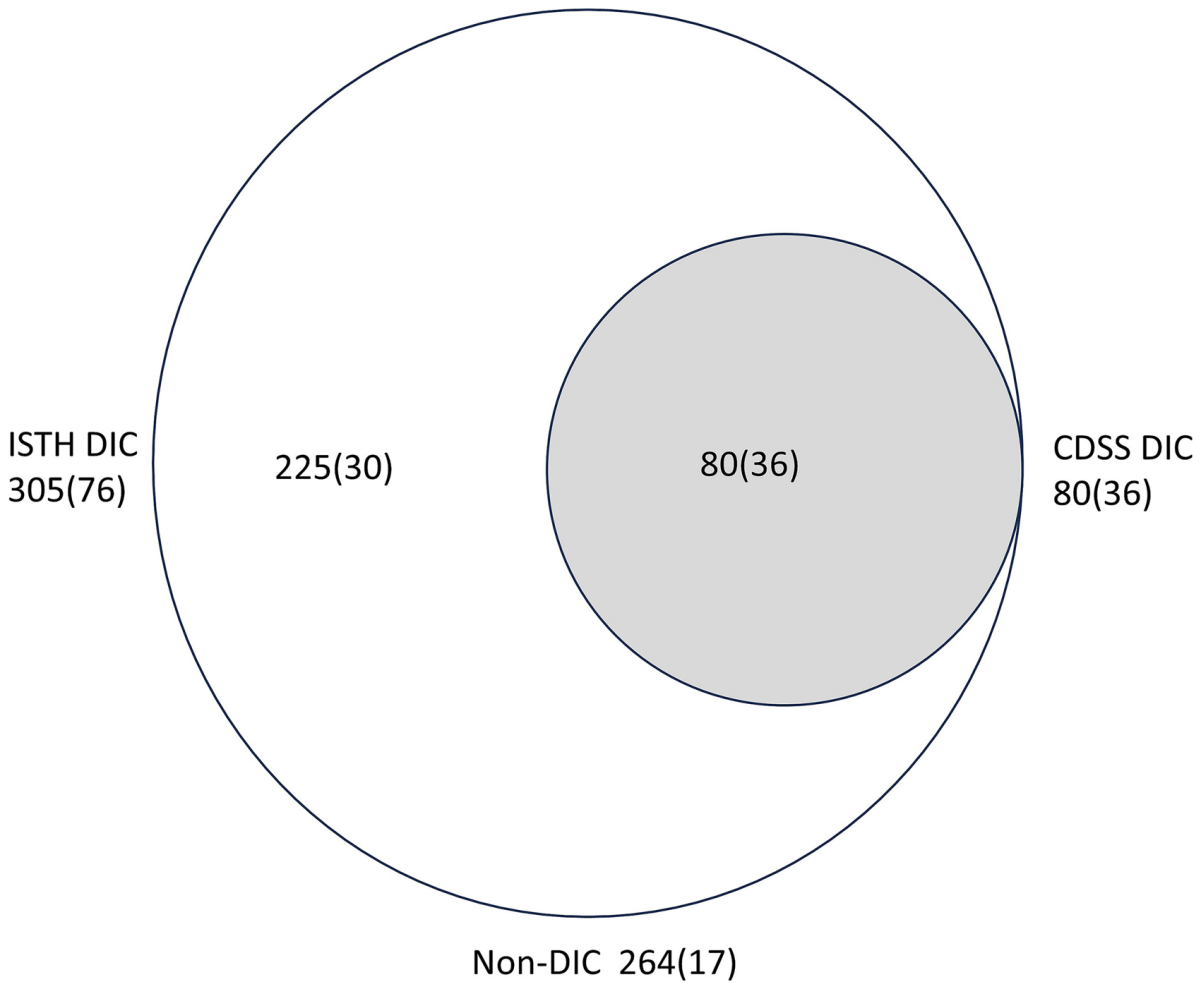
Distribution of patients following liver transplantation according to the two diagnostic criteria. Comparison between the International Society on Thrombosis and Hemostasis (ISTH) and Chinese DIC scoring system (CDSS). Numbers in parentheses are of non-survivors.

Patients with CDSS scores had more severe acute kidney injury, infection, and lymphocytopenia compared to those with ISTH scores. They also had higher mortality rates at 28, 60, and 90 days, longer hospital stays, and shorter survival times. The differences were statistically significant (*p* < 0.05) (Tables 1, 2 and Figure 2).

**Table 2:** Outcomes on CDSS criterion vs. ISTH criterion for disseminated intravascular coagulation in patients following liver transplantation.

	Total (*n* = 569)	CDSS score		ISTH score	
		Non-DIC (*n* = 489)	DIC (*n* = 80)	Non-DIC (*n* = 264)	DIC (*n* = 305)
AKI (%)	136 (23.9)	100 (20.4)	36 (45.0)^a^	34 (12.9)	102 (33.4)^a^
Infection (*n*, %)	207 (36.4)	158 (32.3)	49 (61.3)^a,b^	58 (22.0)	149 (48.9)^a,b^
Lymphocytopenia (*n*, %)	199 (35.2)	162 (33.3)	37 (46.8)^a^	77 (29.3)	122 (40.3)^a^
28-day mortality (%)	61 (10.7)	35 (7.2)	26 (32.5)^a,b^	13 (4.9)	48 (15.7)^a,b^
60-day mortality (*n*, %)	79 (13.9)	44 (9.0)	35 (43.8)^a,b^	17 (6.4)	62 (20.3)^a,b^
90-day mortality (*n*, %)	83 (14.6)	47 (9.6)	36 (45.0)^a,b^	17 (6.4)	66 (21.6)^a,b^
Survival time (d) (median [Q1, Q3])	1,058 [434, 1802]	1,110 [530, 1861]	438 [10, 1,188]^a,b^	1,162 [603, 1801]	944 [218, 1838]^a,b^
ICU time (d) (median [Q1, Q3])	3 [2, 4]	2 [2, 4]	5 [3, 11]^a,b^	2 [1, 3]	3 [2, 6]^a,b^

DIC, disseminated intravascular coagulation; CDSS, Chinese DIC Scoring System; ISTH, International Society on Thrombosis and Hemostasis; AKI, acute kidney injury. ^a^*p* < 0.05 between DIC and non-DIC; ^b^*p* < 0.05 between CDSS DIC and ISTH DIC.

### Association between CDSS and ISTH and illness severity score

**Figure 3:**
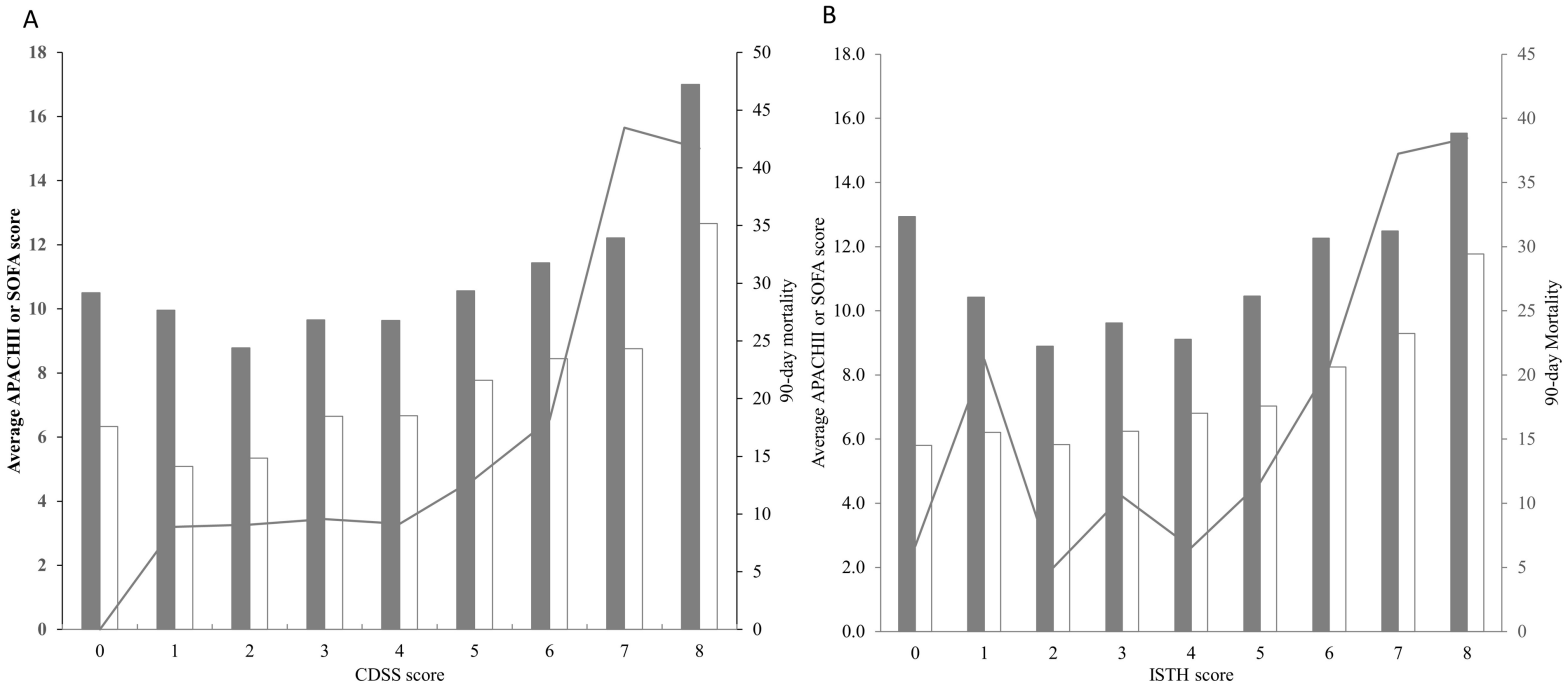
**(A)** Correlations between the CDSS score and the APACHE II score (gray bars), the SOFA score (white bars) as well as the 90-day all-cause mortality (lines). **(B)** Correlations between the ISTH score and the APACHE II score (gray bars), the SOFA score (white bars) as well as the 90-day all-cause mortality (lines).

To compare the correlation between the two scoring methods and the severity of the disease, we compared the CDSS DIC score and the ISTH DIC score with the APACHE II score and the SOFA score, respectively. The results showed that the Spearman’s rank correlation coefficients between the CDSS DIC score and the APACHE II score, as well as the SOFA score, were 0.217 and 0.422, respectively (both *p* < 0.001). Similarly, the Spearman’s rank correlation coefficients between the ISTH DIC score and the APACHE II score, as well as the SOFA score, were 0.19 and 0.371, respectively (both *p* < 0.001) (Figure 3).

### Prognostic value of the CDSS and ISTH criteria

The impact of CDSS and ISTH scoring systems on the prognostic value of all-cause mortality was evaluated using ROC curve analysis. The results of the analysis are presented below:

In the ISTH scoring system, the sensitivity of all-cause mortality at 28 days was 50.77%, the specificity was 88.89%.The area under the AUC curve was 74.1% and odds ratio (95% CI): 3.597 (1.901–6.806). The sensitivity of all-cause mortality at 60 days was 65.82%, the specificity was 73.47%.The area under the AUC curve was 73.2% and odds ratio (95% CI): 3.95 (2.25–6.935). The sensitivity of all-cause mortality at 90 days was 64.29%, specificity was 73.61%. The area under the AUC curve was 73.6% and odds ratio (95% CI): 3.95 (2.25–6.935).

In the CDSS scoring system, the sensitivity for 28-day all-cause mortality was 64.61% and the specificity was 73.62%. The area under the AUC curve was 73.9%, and the odds ratio (95% CI) was 6.228 (3.475–11.1). For 60-day all-cause mortality, the sensitivity was 64.55% and the specificity was 74.70%. The area under the AUC curve was 73.1%, and the odds ratio (95% CI) was 7.719 (4.492–13.266). Finally, for 90-day all-cause mortality, the sensitivity was 63.09% and the specificity was 74.85%. The area under the AUC curve was 73.4%, and the odds ratio (95% CI) was 7.582 (4.435–12.963).

**Figure 4:**
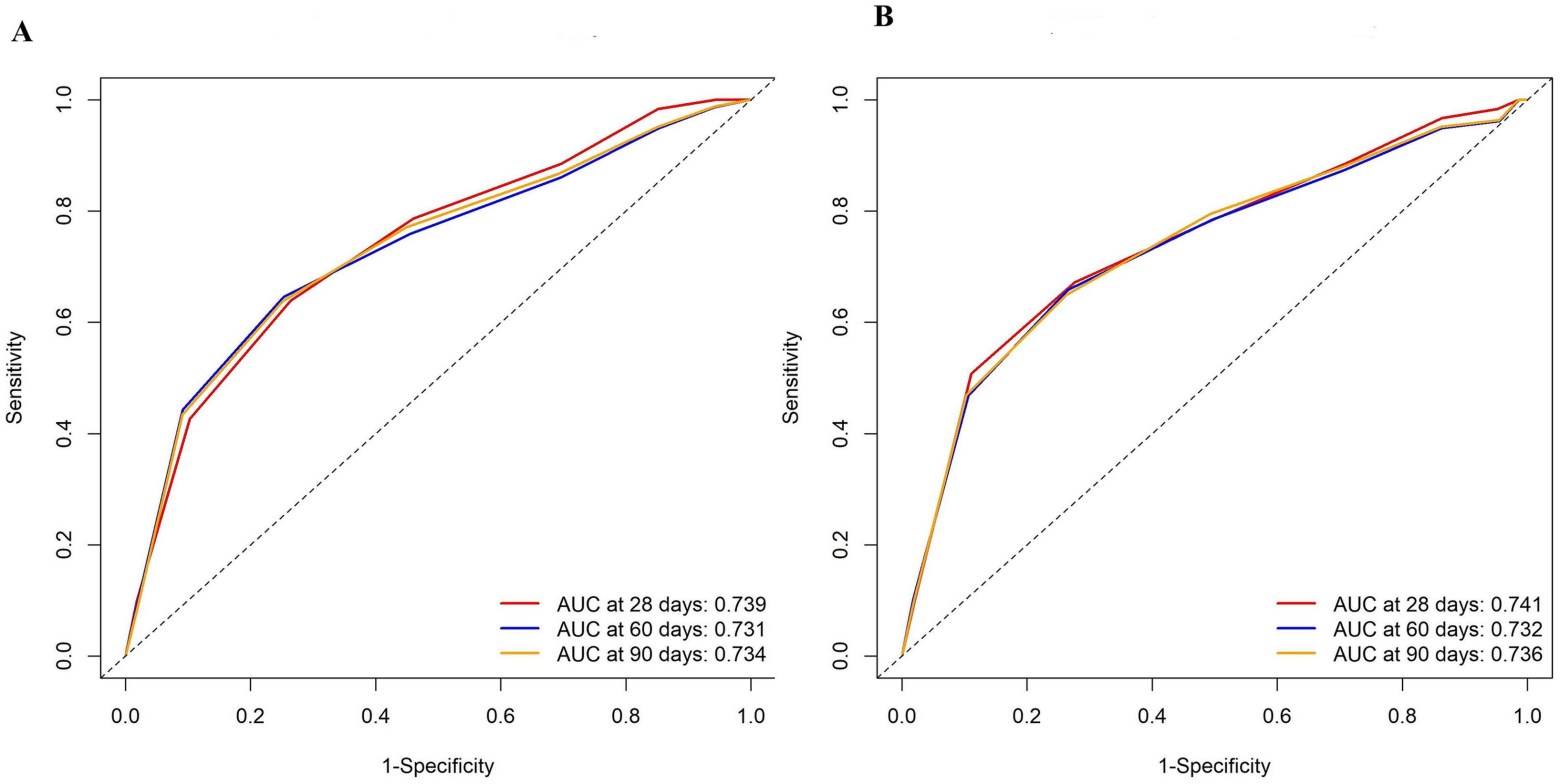
Receiver operating characteristic (ROC) curves with associated AUC of prediction models with the CDSS score **(A)** or the APACHE II score **(B)** to predict mortality at 28,60, and 90 days.

Two types of integration results were summarized, showing higher odds ratios (OR) for 28-day mortality in patients (CDSS: 6.228; ISTH: 3.597; *p* < 0.05). The ORs for predicting mortality with 60-day (7.719 vs. 3.95) and 90-day (7.582 vs. 3.95) criteria with CDSS were higher than those with ISTH (p < 0.05) (Table 3). Additionally, we compared the ROC curve assessment of 28-day, 60-day, and 90-day mortality in patients with DIC using both the APACHEII score and CDSS scoring method. The results revealed a similar area under the curve for both approaches (Figure 4).

**Table 3:** Prognostic value of ISTH and CDSS scoring systems.

	CDSS score	ISTH score
28-day mortality		
Sensitivity	64.61%	50.77%
Specificity	73.62%	88.89%
AUC	0.739	0.741
OR (95%CI)^a,#^	6.228 (3.475–11.164)	3.597 (1.901–6.806)
60-day mortality		
Sensitivity	64.55%	65.82%
Specificity	74.70%	73.47%
AUC	0.731	0.732
OR (95%CI)^a,#^	7.719 (4.492–13.266)	3.95 (2.25–6.935)
90-day mortality		
Sensitivity	63.09%	64.29%
Specificity	74.85%	73.61%
AUC	0.734	0.736
OR (95%CI)^a,#^	7.582 (4.435–12.963)	3.95 (2.25–6.935)

^aAdjusted gender and age.^

^#p < 0.001 for each OR.^

## Discussion

The mainstay of treatment for patients with end-stage liver disease (ESLD) remains LT, which is typically characterized by coagulation abnormalities. The evaluation of the coagulation status is of paramount importance for liver and biliary surgeons, as well as transplant intensive care physicians. Unfortunately, there is currently no scoring system tailored for the assessment of clotting disorders in liver transplant recipients. The results showed the prevalence of DIC is high among patients undergoing LT. Moreover, in liver transplant recipients with DIC, the CDSS score demonstrates superior prognostic value compared to the ISTH score.

Currently, the commonly used diagnostic criteria for DIC scoring systems include the international society of thrombosis and hemostasis Dominant DIC standard (ISTH standard) (9), the Japanese ministry of health and welfare Standard (JMHW standard) (12), and the Japanese association for acute medicine Standard (JAAM standard) (13, 15). Many prospective studies have compared their diagnostic efficacy, but the results remain controversial. While all three criteria aim to identify DIC based on comprehensive clinical manifestations and laboratory indicators, they differ in their emphasis on clinical and laboratory examination features. Additionally, the diagnostic critical values for the same coagulation item index are slightly different across criteria, potentially leading to varying sensitivity and specificity for DIC diagnosis (16, 17). The ISTH proposes scoring criteria presupposing the presence of a known underlying disease associated with DIC. The scoring system primarily includes platelets, PT, fibrinogen, and fibrinogen, with a score ≥ 5 indicative of dominant DIC. Some studies have revealed a strong correlation between ISTH score and mortality, with a one-point increase in score associated with a 1.25 point increase in 28-day risk of death. Consequently, the 2011 guidelines of the Italian Society of Thrombosis and Hemostasis recommend using the ISTH scoring system for DIC diagnosis, as it is the most widely used internationally (4, 18–20). However, the ISTH system has its limitations, such as an excessive focus on platelet counts, potentially leading to overdiagnosis. Moreover, the ISTH diagnosis in non-hematological diseases does not encompass typical clinical features.

In 2014, China referenced the three major foreign scoring systems and integrated clinical experience to optimize the CDSS scoring system. This involved the adoption of rigorous statistical methods and conducting multi-center, large-sample, retrospective, and prospective studies to establish a scientific and reliable quantitative diagnostic tool for DIC (21). The CDSS score offers several advantages: (1) It emphasizes the significance of underlying disease and clinical manifestations, including the presence of primary disease causing DIC, clinical manifestations, Platelets (PLT), D-Dimer, prothrombin time (PT) and activated partial thromboplastin time (APTT) and fibrinogen (Fib). DIC can be divided into high coagulation stage, waste low coagulation stage, secondary high fibrinolysis stage and organ failure stage. In clinical practice, it has been observed that a small number of patients did not display obvious DIC-related abnormalities when DIC occurred. Considering the underlying disease as the overall reference for the diagnosis of DIC would enhance the sensitivity of DIC diagnosis. (2) Previous studies have revealed a dynamic decrease in platelets, with DIC patients increasing by nearly 21%. The CDSS score underscores the importance of monitoring the dynamic decline of platelets to aid in the early diagnosis of DIC (16). (3) Previous studies have also demonstrated the significant impact of D-D polymers on the diagnosis of DIC. DIC is characterized by secondary fibrinolysis, and D-D polymers are specific markers of secondary fibrinolysis. Therefore, the inclusion of D-D polymers in CDSS enhances its diagnostic value, with its specificity is better than FDP (17, 22). The level of coagulation factor is related to hemostasis. In the development of DIC, the continuous activation of coagulation factor and the subsequent depletion lead to reduced levels of coagulation factor, resulting in prolonged PT and APTT, which are highly correlated with the severity of DIC. As a result, the CDSS scoring system incorporates APTT and PT in the overall coagulation profile to improve the sensitivity of DIC diagnosis. Considering the aforementioned advantages of CDSS and taking into account the racial characteristics of blood coagulation, this study aims to compare the commonly used international ISTH score and the domestic CDSS score in assessing the prognosis of LT patients with blood coagulation dysfunction. The goal is to identify a more effective assessment tool for the prognosis of Asian ethnic liver transplantation patients with blood coagulation dysfunction.

Disseminated intravascular coagulation, a severe complication following liver transplantation, arises from the significantly reduced coagulation and fibrinolytic functions in patients with end-stage liver failure before and after surgery. The competition between acute inflammation and coagulation/fibrinolysis results in thrombotic microangiopathy and systemic inflammatory response syndrome, contributing to a poor prognosis (7, 23–25). Our study found that patients with CDSS scores had a higher incidence of acute kidney injury (AKI), infection, lymphocytopenia, and mortality compared to patients with ISTH scores. After adjusting for age and sex, the CDSS score demonstrated better prognostic value than the ISTH score in patients with LT-associated DIC. This study also observed that DIC cases diagnosed by ISTH criteria encompassed all cases diagnosed by CDSS criteria. Our study demonstrates that the odds ratio (OR) value of the CDSS score is higher than that of ISTH in assessing patient death outcomes. Taking 28-day fatality rate as an example, for every 1 point increase in CDSS score, the fatality rate of patients increased by 6.228 times, while for every 1 point increase in ISTH score, the fatality rate of patients increased by 3.597 times, indicating that the CDSS score effect value was higher, it can judge the death prognosis of patients more timely. Specifically, according to the diagnostic criteria of CDSS, the mortality rate of LT patients with DIC is higher, indicating that the CDSS score can better predict the death prognosis of these patients, leading to a reduction in false-positive rates and a more efficient use of medical resources (21, 26).

It is well known that the APACHE II score and SOFA score are important tools for evaluating the severity of patients (27–29). In our study, CDSS scores and ISTH scores exhibited a general rise in tandem with the escalation of APACHE II scores and SOFA scores. ROC curve analysis revealed that CDSS score demonstrated comparable predictive ability to the APACHEII score for 28-day, 60-day and 90-day mortality in patients, which was consistent with previous studies (30, 31). It is further indicates that CDSS score can effectively mirror disease severity and possesses substantial prognostic value.

The present study also exhibits certain limitations. Being a single-center retrospective study with a limited number of cases, the generalizability of the findings may be constrained. Continuing investigations seek to expand upon these preliminary results by involving larger and more diverse patient populations across multiple centers.

## Conclusion

The occurrence rate of coagulation dysfunction is high in patients undergone LT. The CDSS score has better prognostic value than the ISTH score in DIC patients with LT in China. A prospective randomized controlled study should be designed to further assess the relevant findings.

## Data Availability

The original contributions presented in the study are included in the article/Supplementary material, further inquiries can be directed to the corresponding authors.
