# Supplementary material for: CDSS score is favorable to ISTH score on outcomes for disseminated intravascular coagulation in patients with liver transplantation: a retrospective cohort study
Source: Front Med (Lausanne). 2025 Apr 30;12:1514139. doi: 10.3389/fmed.2025.1514139 (PMC12076933; doi:10.3389/fmed.2025.1514139)
Supplement: Supplementary file 1 [file Table_1.pdf]

Supplementary Table1 Criteria for three DIC scoring systems

Item	Point	ISTH criteria	CDSS criteria
Clinical presentation	1	_	abnormal bleeding*
			unexplained organ failure
			shock or microcirculatory disorder independent of original disease
			（meet anyone 1 point）
PT, PT ratio,		Prolongation of PT	Prolongation of PT and APTT
Prolongation of PT	0	＜3s	PT＜3s and APTT＜10s
Prolongation of APPT	1	⩾3s	PT⩾3s or APTT⩾10s
	2	⩾6s	PT⩾6s
Fibrin-related marker		D-dimer (ug/mL)	D-dimer (μg/mL)
	0	＜0.5	＜5
	1	-	-
	2	0.5-5	5–9
	3	⩾5	⩾9
Fibrinogen level (g/L)	0	>1	＞1
	1	⩽1	⩽1
	2	-	-
Platelet counts (x10^3/μL)	0	＞100	＞100
	1	50-100	80-100 or≥50% decrease within 24 hrs. (#) < 80
	2	< 50	
Diagnosis of DIC		≥5 points	≥7 points
